# History of Obstructive Pyelonephritis Treated Without Drainage as a Risk Factor for Febrile Urinary Tract Infection After Ureteroscopic Lithotripsy: A Retrospective Study From Three Institutions

**DOI:** 10.7759/cureus.89377

**Published:** 2025-08-04

**Authors:** Takayuki Ueda, Masato Yanagi, Masato Nagasawa, Jun Akatsuka, Shuichi Osawa, Ryoji Kimata, Tsutomu Hamasaki, Taiji Nishimura, Yukihiro Kondo

**Affiliations:** 1 Urology, Aidu Chuo Hospital, Fukushima, JPN; 2 Urology, Nippon Medical School Hospital, Tokyo, JPN; 3 Urology, Heisei-Tateishi Hospital, Tokyo, JPN; 4 Urology, Nippon Medical School Musashikosugi Hospital, Kawasaki, JPN

**Keywords:** drainage, febrile urinary tract infection, obstructive pyelonephritis, ureteral stent, ureteroscopic lithotripsy

## Abstract

Purpose: Preoperative obstructive pyelonephritis (OP) increases the risk of febrile urinary tract infection (fUTI) after ureteroscopic lithotripsy (URSL). This study aimed to investigate the effect of a history of OP treated without drainage on post‑URSL fUTI.

Methods: We retrospectively reviewed the medical records of 343 consecutive patients who underwent URSL at three institutions between January 2021 and April 2024. Risk factors for post‑URSL fUTI were analyzed, and frequencies were compared among patients with a history of OP treated without drainage, those with a history of OP treated with ureteral stent (US) placement, and those without a history of OP.

Results: Of the 343 patients, 29 (8.5%) developed post‑URSL fUTI. Multivariate logistic regression analysis revealed that a history of OP (p < 0.001) and preoperative positive urine culture (p = 0.043) were independent risk factors for post‑URSL fUTI. The incidence of post‑URSL fUTI was significantly higher in patients with OP treated with drainage than in those without a history of OP (p < 0.001). Moreover, the incidence of post‑URSL fUTI in patients with OP treated without drainage was significantly higher than that in patients treated with US placement (p = 0.030).

Conclusion: In this study, the incidence of post‑URSL fUTI in patients with OP treated without drainage was significantly higher than that in those treated with US placement. A history of OP treated without drainage might represent the highest risk factor for post‑URSL fUTI. Therefore, calculous pyelonephritis probably should be managed with drainage to mitigate this risk.

## Introduction

Ureteroscopic lithotripsy (URSL) is the standard surgical procedure for upper urinary tract stones [1]. Febrile urinary tract infection (fUTI) after URSL, which can be fatal if it progresses to septic shock, is a major concern for patients undergoing the procedure, with an incidence rate of 4.0-11.5% [2-9]. A history of obstructive pyelonephritis (OP) has been reported as a risk factor for post‑URSL fUTI [10,11]. In addition, among patients with a history of OP, a prolonged ureteral stent (US) placement period has been identified as a risk factor for post‑URSL fUTI [12]. However, the impact of a history of OP treated without drainage on post‑URSL fUTI has not yet been studied. OP is generally an indication for drainage, and the mortality rate is significantly lower in patients who undergo drainage within two days of onset compared with those treated after two days [13]. Therefore, early drainage is essential in the management of OP. In clinical practice, however, OP is sometimes managed without drainage for various reasons. In our institutions, we occasionally encountered cases of calculous pyelonephritis that responded significantly to initial antimicrobial therapy initiated by non‑urologists. In such cases, the patient’s condition sometimes improved, and the urologist’s recommendation for ureteral stenting was declined. We defined OP treated without drainage as OP managed without US placement or nephrostomy placement. This study aimed to investigate the impact of a history of OP treated without drainage on post‑URSL fUTI. To our knowledge, no previous studies have focused on OP treated without drainage. This is the first study to analyze patients with a history of OP who were managed without drainage.

A pilot study of this research was previously posted to the Research Square preprint server on January 3, 2024.

## Materials and methods

Patient selection

We retrospectively reviewed consecutive patients treated with URSL at three institutions, Aidu Chuo Hospital, Nippon Medical School Musashikosugi Hospital, and Heisei‑Tateishi Hospital, between January 2021 and April 2024, using a surgical database. Cases involving second or subsequent URSL for the same stone, staghorn calculi, simultaneous bilateral URSL, concurrent surgery with other procedures, immediate URSL for OP, nephrostomy placement, or insufficient data were excluded. Finally, 343 patients who underwent URSL were included in the study.

Clinical data

Clinical information was evaluated, including age, sex, body mass index (BMI), Eastern Cooperative Oncology Group performance status (ECOG PS), history of diabetes mellitus (DM), history of stone surgery, presence or absence of hydronephrosis at initial diagnosis, history of OP, presence or absence of preoperative ureteral stent (US) placement, preoperative US placement period, laterality and location of stones, stone size, multifocality of stones, preoperative urine culture, use of flexible ureteroscopy, operative time, and outcomes, including the stone‑free rate and post‑URSL fUTI. Patients with preoperative US placement included those who required pre‑stenting for ureteral stenosis unrelated to OP. Preoperative OP was diagnosed and treated by the attending physician based on clinical findings, including computed tomography (CT) and laboratory data such as bacteriuria and leukocyturia. Post‑URSL fUTI was defined as body temperature >38 °C within 3 days of URSL, and septic shock was defined as a case requiring noradrenaline administration.

Surgical procedure

URSL was performed with the patient in the lithotomy position under general or spinal anesthesia. A rigid ureteroscope (OES Pro; Olympus, Hamburg, Germany) and a flexible ureteroscope (URF‑P5; Olympus, Hamburg, Germany) were used. A flexible ureteroscope equipped with a ureteral access sheath was employed during the procedure. Stones were fragmented or dusted using a Holmium:YAG laser (VersaPulse; Lumenis, Tel Aviv, Israel) and collected using a nitinol stone retrieval basket (Escape; Boston Scientific, Natick, MA, USA). To prevent post‑URSL fUTI, 1.0 g of cefmetazole or 2.0 g of piperacillin was administered immediately before and after surgery, and again in the morning and evening of the following day. In patients with a positive preoperative urine culture, antibiotics were administered according to the same schedule based on drug sensitivity. Additional preoperative and postoperative antibiotic administration was at the discretion of the attending physician. Several surgeons, including non‑expert urologic fellows, performed URSL. Four expert surgeons (MN, HK, RK, and SO), each with experience in >300 URSL cases, performed or supervised all procedures. Stone‑free status was defined as no remaining stones ≥4 mm on the operated side. Postoperative ureteral stenting was performed for all patients and was generally removed later than one week after URSL.

Statistical analysis

Statistical analyses were performed using IBM SPSS Statistics for Windows, Version 29 (Released 2022; IBM Corp., Armonk, New York). Statistical significance was set at p < 0.05. Categorical variables were compared using the chi‑square test or Fisher’s exact test. Continuous variables were compared using the t‑test or Mann‑Whitney U test, depending on the results of the one‑sample Kolmogorov‑Smirnov test. Univariate and multivariate analyses were performed using a logistic regression model to identify independent risk factors for post‑URSL fUTI. The cutoff values for BMI and stone size were defined as the medians of these variables in the 343‑patient cohort. The cutoff value for operative time was defined as 90 minutes because operating time ≥90 minutes has been reported as a risk factor for severe adverse events after URSL [11]. We compared the characteristics of patients with a history of OP treated without US placement with those of patients with a history of OP treated with US placement. We also compared the rates of post‑URSL fUTI among patients without a history of OP, those with a history of OP treated without drainage, and those with a history of OP treated with US.

## Results

Table 1 presents the demographics of the 343 patients who underwent URSL (216 (63.0%) men and 127 (37.0%) women; median age, 60 years; range, 18-93 years). The median BMI was 24.3 kg/m² (range, 15.0-41.0), and 98 (28.6%) patients had a history of OP. Renal and ureteral stones were observed in 82 (23.9%) and 261 (76.1%) patients, respectively. The median stone size was 8 mm (range, 3-25 mm). In all cases in which a US was placed for OP, the stent was placed within three days of OP onset. The median preoperative US placement period was five weeks (range, 2-16 weeks). The median operative time was 44 minutes (range, 12-199 minutes). The stone‑free rate of URSL was 94.5%. Post‑URSL fUTI occurred in 29 (8.5%) of the 343 patients, and one of these 29 patients developed septic shock. No cases of severe intraoperative ureteral injuries were identified.

**Table 1:** Patient demographics BMI: body mass index; IQR: interquartile range; ECOG PS: Eastern Cooperative Oncology Group performance status; DM: diabetes mellitus; OP: obstructive pyelonephritis; US: ureteral stent; URSL: ureteroscopic lithotripsy; fUTI: febrile urinary tract infection.

Variables	Categories	Values, n (%) (N=343)
Age (years); median (IQR)		60 (51–71)
Sex	Male	216 (63.0)
	Female	127 (37.0)
BMI (kg/m^2^); median (IQR)		24.3 (22.1–27.2)
ECOG PS	0	316 (92.1)
	1	20 (5.8)
	2	4 (1.2)
	3	2 (0.6)
	4	1 (0.3)
History of DM	Yes	17 (5.0)
	No	326 (95.0)
Prior stone surgery	Yes	15 (4.4)
	No	328 (95.6)
Grade of hydronephrosis	0	76 (22.2)
	1	93 (27.1)
	2	116 (33.8)
	3	47 (13.7)
	4	11 (3.2)
History of OP	Yes	98 (28.6)
	No	245 (71.4)
Preoperative placement of US	Yes	113 (32.9)
	No	230 (67.1)
US placement period (weeks); median (IQR)		5 (4–7)
Laterality	Right	146 (42.6)
	Left	197 (57.4)
Location of stones	R2	37 (10.7)
	R3	45 (13.2)
	U1	143 (41.7)
	U2	52 (15.2)
	U3	66 (19.2)
Stone size (mm); median (IQR)		8 (6–11)
Multifocality	Single	285 (83.1)
	Multiple	58 (16.9)
Preoperative urine culture	Positive	50 (14.6)
	Negative	293 (85.4)
Prophylactic antibiotics	Yes	9 (2.6)
	No	334 (97.4)
Use of flexible ureteroscopy	Yes	185 (53.9)
	No	158 (46.1)
Operating time (min); median (IQR)		44 (30–63)
Stone-free status	Yes	324 (94.5)
	No	19 (5.5)
Post-URSL fUTI	Yes	29 (8.5)
	No	314 (91.5)
Stone type	Calcium Oxalate	326 (95.0)
	Others	17 (5.0)

The univariate logistic regression analysis revealed that ≥ 65-years old (p=0.007), female (p<0.001), ECOG PS ≥2 (p=0.023), History of OP (+) (p<0.001), preoperative placement of US (p=0.003), and positive preoperative urine culture (p<0.001) were risk factors of post-URSL fUTI. Multivariate logistic regression analysis revealed that history of OP (p<0.001; OR, 10.63; 95% CI, 3.19-35.38) and preoperative positive urine culture (p=0.043; OR, 2.88; 95% CI, 1.03-8.03) were independent risk factors for post-URSL fUTI (Table 2).

**Table 2:** Univariate and multivariate logistic regression analyses of risk factors for fUTI after URSL *p<0.05 OR: odds ratio; CI: confidence interval; BMI: body mass index; ECOG PS: Eastern Cooperative Oncology Group performance status; DM: diabetes mellitus; OP: obstructive pyelonephritis; US: ureteral stent; URSL: ureteroscopic lithotripsy.

Variables	Univariate			Multivariate		
	OR	95% CI	P-value	OR	95% CI	P-value
≥65 years old	2.91	1.31–6.47	0.007*	-	-	-
Female	5.15	2.21–12.02	<0.001*	-	-	-
BMI>24.3 kg/m^2^	0.58	0.27–1.27	0.167	-	-	-
ECOG PS ≥2	7.13	1.61–31.52	0.023*	-	-	-
DM (+)	2.47	0.67–9.16	0.165	-	-	-
Prior stone surgery (+)	1.72	0.38–8.00	0.368	-	-	-
Hydronephrosis grade ≥3	1.47	0.65–3.33	0.960	-	-	-
History of OP (+)	9.84	4.05–23.94	<0.001*	10.63	3.19–35.38	<0.001*
Preoperative placement of US	3.22	1.48–7.00	0.003*	0.42	0.13–1.34	0.142
Right stone	0.67	0.31–1.43	0.300	-	-	-
Renal stone	1.68	0.75–3.77	0.221	-	-	-
Stone size on imaging >8mm	1.23	0.58–2.64	0.590	-	-	-
Multiple stones	1.64	0.67–4.04	0.299	-	-	-
Preoperative urine culture (+)	7.21	3.22–16.15	<0.001*	2.88	1.03–8.03	0.043*
Prophylactic antibiotics (–)	3.25	0.64–16.42	0.171	-	-	-
Use of flexible ureteroscopy on URSL (+)	1.23	0.57–2.66	0.596	-	-	-
Operating time ≥90min	1.05	0.30–3.68	0.935	-	-	-

**Figure 1:**
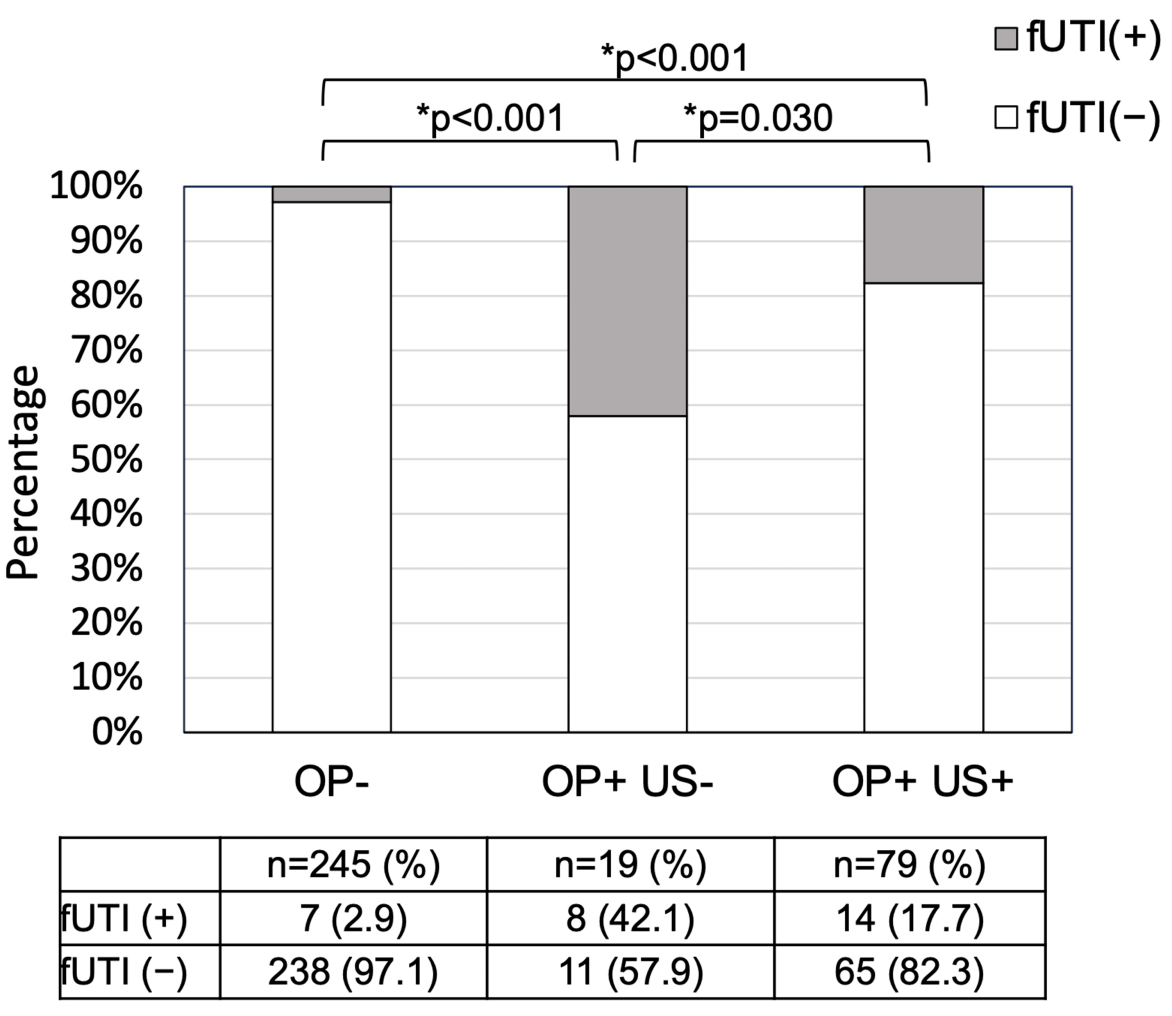
Comparison of rate of post-URSL fUTI between patients without a history of OP, patients with a history of OP treated without US (drainage), and patients with a history of OP treated with US Comparison was performed using the chi-square test. URSL, ureteroscopic lithotripsy; fUTI, febrile urinary tract infection; OP, obstructive pyelonephritis; US, ureteral stent; (-), absent; (+), present; *p<0.05. URSL: ureteroscopic lithotripsy; fUTI: febrile urinary tract infection; OP: obstructive pyelonephritis; US: ureteral stent.

Of the 98 patients with a history of OP, 19 (19.4%) underwent treatment without drainage. Patients treated with drainage for OP showed significantly higher rates of positive urine cultures at OP (p<0.001) and positive preoperative urine cultures (p=0.004) than those treated without drainage (Table 3). All patients who experienced OP with septic shock underwent US placement (Table 3). The incidence of post-URSL fUTI was markedly higher in patients with OP treated with drainage than in those without a history of OP (p<0.001) (Figure 1). Similarly, patients with a history of OP who were treated without drainage exhibited a significantly higher incidence of post-URSL fUTI than those without a history of OP (p<0.001) (Figure 1). Additionally, the incidence of post-URSL fUTI in patients with OP treated without drainage was significantly higher than that in those treated with US placement (p=0.030) (Figure 1). Furthermore, the rates of positive urine culture at OP (p<0.001) and positive preoperative urine culture (p=0.004) were significantly lower in patients with a history of OP who were treated without drainage than in those treated with US placement (Table 3).

**Table 3:** Characteristics of patients with a history of OP treated without US (drainage), and patients with a history of OP treated with US Categorical variables were compared using the chi-square test or Fisher’s exact test. Continuous variables were compared using either the t-test or Mann–Whitney U test, depending on the results of the one-sample Kolmogorov–Smirnov test. *p<0.05. OP: obstructive pyelonephritis; US: ureteral stent; BMI: body mass index; ECOG PS: Eastern Cooperative Oncology Group performance status; DM: diabetes mellitus; URSL: ureteroscopic lithotripsy; fUTI: febrile urinary tract infection.

Variables	OP (+) US (-)	OP (+) US (+)	P-value	Statistical methods
n	19	79		
Age (years old)	64 (51–68)	72 (60–78)	0.105	Mann–Whitney U test
Male	14 (73.7)	50 (63.3)	0.385	Chi-square test
BMI≥24.3 kg/m^2^	24.6 (22.8–27.1)	22.6 (19.9–26.2)	0.063	Mann–Whitney U test
ECOG PS ≥2	0 (0.0)	7 (8.9)	0.340	Fisher’s exact test
DM (+)	2 (10.5)	10 (12.7)	1.000	Fisher’s exact test
Prior stone surgery (+)	2 (10.5)	8 (10.1)	1.000	Fisher’s exact test
Grade of hydronephrosis ≥3	2 (10.5)	18 (22.8)	0.346	Fisher’s exact test
History of OP with septic shock (+)	0 (0.0)	13 (16.5)	0.067	Fisher’s exact test
Positive urine culture at OP	5 (26.3)	55 (69.6)	<0.001*	Fisher’s exact test
Right stone	7 (36.8)	36 (45.6)	0.489	Chi-square test
Renal stone	4 (21.1)	10 (12.7)	0.463	Fisher’s exact test
Stone size on imaging (mm)	7 (6-10)	7 (5-10)	0.652	Chi-square test
Multiple stones	1 (5.3)	21 (26.6)	0.064	Fisher’s exact test
Preoperative urine culture (+)	3 (15.8)	43 (54.4)	0.004*	Fisher’s exact test
Prophylactic antibiotics	2 (10.5)	7 (8.9)	1.000	Fisher’s exact test
Use of flexible ureteroscopy on URSL (+)	10 (52.6)	51 (64.6)	0.325	Mann–Whitney U test
Operating time (min)	40 (24–62)	49 (34–80)	0.118	Mann–Whitney U test
Post-URSL fUTI (+)	8 (42.1)	14 (17.7)	0.030*	Chi-square test

## Discussion

A history of OP has been identified as a risk factor for post‑URSL fUTI [10,11], while prolonged operating time has also been associated with this complication [11]. In this study, a history of OP was a risk factor for post‑URSL fUTI. However, prolonged operating time was not a significant risk factor for post‑URSL fUTI. Our results suggest that patient factors, such as a history of OP, were more strongly associated with post‑URSL fUTI than surgical factors, such as prolonged operating time. Furthermore, the incidence of post‑URSL fUTI was significantly higher in patients with a history of OP treated without drainage than in those treated with US placement. To the best of our knowledge, this is the first study to explore the effects of treatment without drainage for OP on subsequent URSL outcomes.

Bacteria can colonize stones in the urinary tract [14,15]. We hypothesized that in patients with a history of OP, both bacteria colonizing stones and those on the inserted US would be significant contributors to post‑URSL fUTI. However, patients with a history of OP who were treated without drainage also had a significantly higher incidence of post‑URSL fUTI than those without a history of OP (Figure 1). These results suggest that a history of OP, regardless of US placement, is a risk factor for post‑URSL fUTI. The main cause of post‑URSL fUTI was suspected to be stone‑colonizing bacteria in patients with a history of OP, which were released into the urine when the stones were crushed during URSL. Furthermore, the incidence of post‑URSL fUTI was significantly higher in patients with a history of OP treated without drainage than in those treated with US placement. One possible explanation is that stent implantation increases urine flow, potentially aiding in the drainage of bacteria colonizing stones, thus reducing the bacterial load on the stones. Future studies should compare bacterial adherence to stones between patients with a history of OP treated without drainage and those treated with drainage alone. A recent systematic review revealed that prophylactic antibiotics have some effect [16]. The incidence of post‑URSL fUTI has been documented to range from 4.0-11.5% [2-9]. In our institutions, the incidence of post‑URSL fUTI was 8.5%, which is consistent with reports from other institutions. This rate could potentially be slightly lowered through routine drainage in cases of OP and additional prophylactic antibiotic administration in patients with a history of OP, irrespective of US placement.

The present study has several limitations. This retrospective study was conducted at three institutions with a small cohort. With only one case of postoperative sepsis, the present study lacks the power to analyze risk factors for severe fUTI after URSL. Further prospective studies with larger cohorts are required. Our hospitals are educational institutions; therefore, non‑expert surgeons participated in URSL procedures. Therefore, a surgeon selection bias may exist. However, three expert surgeons supervised all URSL procedures and performed them when necessary. No cases of severe intraoperative ureteral injury were identified in the present study. Therefore, we believe that the participation of non‑expert surgeons has little impact on URSL quality.

In the present study, the attending physicians decided on the preoperative treatment for OP, and no clear criteria existed for the indications of US placement for OP. The present study lacked detailed data on the severity of OP, such as the SOFA score. Moreover, the lack of standardized criteria for drainage introduces the possibility that patients treated without drainage had less severe OP. Absence of clear criteria for drainage might have some impact on the study results. In the future, it will be necessary to establish a treatment protocol for OP.

In addition, cefmetazole was selected as one of the perioperative prophylactic antibacterial agents, which deviates from the recommendation of the Japanese guideline [17] and the AUA guideline. Therefore, cefmetazole used in this study is not recommended, but its effectiveness is similar to that of second‑generation cephalosporins, which are appropriate antibiotics for URSL. This might affect the results of the present study. Further studies, including a cohort with an optimal perioperative prophylactic antibacterial agent, are required. The AUA statement on antibiotic prophylaxis is clear that treatment should be undertaken first for elective procedures, and the AUA guidelines on surgical management of stones indicate that drainage should be performed with URSL deferred until later. Contrary to this, our cohort included patients with positive preoperative urine cultures. Furthermore, the population of patients with a history of stone treatment and those with positive preoperative urine culture who received preoperative antibiotics was low. These might affect the results of the present study. Future analyses including these detailed data are needed.

Finally, in this study, irrigation pressure, which could potentially affect post‑URSL fUTI [18,19], was not analyzed. Further research incorporating an analysis of irrigation pressure is warranted.

## Conclusions

A history of OP is a risk factor for post-URSL fUTI. In the present study, the incidence of post-URSL fUTI in patients with OP treated without drainage was significantly higher than that in those treated with US placement. A history of OP treated without drainage might be the highest risk factor for post-URSL fUTI. Therefore, calculous pyelonephritis should be managed using drainage to mitigate this risk.
